# Supplementary material for: Comparison between Experience-Based and Household-Undernourishment Food Security Indicators: A Cautionary Tale
Source: Nutrients. 2020 Oct 29;12(11):3307. doi: 10.3390/nu12113307 (PMC7692183; doi:10.3390/nu12113307)
Supplement: Supplementary file 1 [file nutrients-12-03307-s001.pdf]

Supplementary Materials.

All the questions start with: During the last 3 months, because of lack of money or other resources.

Table S1. ELCSA's questionnaire.

No.	Question in Spanish	Translation to English	Dimension
1	… ¿alguna vez usted se preocupó porque los alimentos no se acabaran en su hogar?	… did you ever worry you may not have enough food at home?	Anxiety - household
2	… ¿alguna vez en su hogar se quedaron sin alimentos?	… has your household ever been left without food?	Food quantity - household
3	… ¿alguna vez en su hogar dejaron de tener una alimentación sana y balanceada?	… has your household ever not had a healthy diet?	Food quantity and quality - household
4	… ¿alguna vez usted o algún adulto en su hogar tuvo una alimentación basada en poca variedad de alimentos?	… have you or another adult in your household ever had a diet based in poor food variety?	Food quality - household
5	… ¿alguna vez usted o algún adulto dejó de desayunar, almorzar o cenar?	… have your or another adult in your household ever not had breakfast, lunch or dinner?	Food quantity/ Coping strategy - adults
6	… ¿alguna vez usted o algún adulto en su hogar comió menos de los debía comer?	… have you or another adult in your household ever eaten less than you should?	Food quantity/ Coping strategy - adults
7	… ¿alguna vez usted o algún adulto en su hogar sintió hambre pero no comió?	… have you or another adult in your household felt hunger but no eaten?	Sensation - adults
8	… ¿alguna vez usted o algún adulto en su hogar solo comió una vez al día o dejó de comer durante todo un día?	… have you or another adult in your household ever only eaten once a day or stopped eating for a whole day?	Food quantity/ Coping strategy – adults
Survey continues only if the household has children (under 18 years)			
9	… ¿alguna vez algún menor de 18 años en su hogar dejó de tener una alimentación saludable y balanceada?	… has anyone under 18 in your household ever stopped having a healthy diet?	Quantity and quality – under 18
10	… ¿alguna vez algún menor de 18 años en su hogar tuvo una alimentación basada en poca variedad de alimentos?	… has anyone under 18 in your household ever had a diet based in poor food variety?	Food quality – under 18
11	… ¿alguna vez algún menor de 18 años en su hogar dejó de desayunar, almorzar o cenar?	… has anyone under 18 in your household ever stopped having breakfast, lunch or dinner?	Food quantity/ Coping strategy – under 18
12	… ¿alguna vez algún menor de 18 años en su hogar comió menos de lo que debía?	… has anyone under 18 in your household ever eaten less than they should?	Food quantity/ Coping strategy – under 18
13	… ¿alguna vez tuvieron que disminuir la cantidad servida en las comidas a algún menor de 18 años en su hogar?	… have you ever had to reduce the quantity of food served to anyone under 18 in your household?	Food quantity/ Coping strategy – under 18
14	… ¿alguna vez algún menor de 18 años en su hogar sintió hambre pero no comió?	… has anyone under 18 in your household ever felt hunger but didn't eat?	Sensation – under 18
15	… ¿alguna vez algún menor de 18 años en su hogar solo comió una vez al día o dejó de comer durante todo un día?	… has anyone under 18 in your household ever only eaten once a day or stopped eating for a whole day?	Food quantity/ Coping strategy – under 18
